# Comparative Pathogenesis of Asian and African-Lineage Zika Virus in Indian Rhesus Macaque’s and Development of a Non-Human Primate Model Suitable for the Evaluation of New Drugs and Vaccines

**DOI:** 10.3390/v10050229

**Published:** 2018-05-01

**Authors:** Jonathan O. Rayner, Raj Kalkeri, Scott Goebel, Zhaohui Cai, Brian Green, Shuling Lin, Beth Snyder, Kimberly Hagelin, Kevin B. Walters, Fusataka Koide

**Affiliations:** 1 Department of Infectious Disease Research, Southern Research Institute, Birmingham, AL 35205, USA; jrayner@southalabama.edu; 2 Department of Microbiology and Immunology, University of South Alabama, Mobile, AL 36688, USA; 3 Department of Infectious Disease Research, Southern Research Institute, Frederick, MD 21701, USA; rkalkeri@southernresearch.org (R.K.); sgoebel@southernresearch.org (S.G.); zcai@southernresearch.org (Z.C.); bgreen@southernresearch.org (B.G.); slin@southernresearch.org (S.L.); bsnyder@southernresearch.org (B.S.); khagelin@southernresearch.org (K.H.); kwalters@southernresearch.org (K.B.W.)

**Keywords:** Zika virus, ZIKV, rhesus macaques, Non-human primates, NHP, infection, natural history, Asian-lineage, African-lineage

## Abstract

The establishment of a well characterized non-human primate model of Zika virus (ZIKV) infection is critical for the development of medical interventions. In this study, challenging Indian rhesus macaques (IRMs) with ZIKV strains of the Asian lineage resulted in dose-dependent peak viral loads between days 2 and 5 post infection and a robust immune response which protected the animals from homologous and heterologous re-challenge. In contrast, viremia in IRMs challenged with an African lineage strain was below the assay’s lower limit of quantitation, and the immune response was insufficient to protect from re-challenge. These results corroborate previous observations but are contrary to reports using other African strains, obviating the need for additional studies to elucidate the variables contributing to the disparities. Nonetheless, the utility of an Asian lineage ZIKV IRM model for countermeasure development was verified by vaccinating animals with a formalin inactivated reference vaccine and demonstrating sterilizing immunity against a subsequent subcutaneous challenge.

## 1. Introduction

Since its introduction into the Americas, Zika virus (ZIKV) has been the subject of a widespread outbreak linked to a number of different fetal abnormalities including congenital microcephaly [1,2,3,4,5,6] and neurological disorders such as Guillain-Barre syndrome (GBS) [7]. Previously, human infections with ZIKV were infrequent or underreported and associated with only mild symptoms including headache, myalgia, rash and a self-limiting fever; thus, no vaccines or therapeutics have been developed against ZIKV despite the fact that the virus has been known since 1947 [8]. Given the magnitude of the recent outbreak and association with significant clinical manifestations, there is a heightened need for medical interventions to prevent or treat ZIKV infections in humans. Concomitant with the need for new vaccines and therapeutics is the need for appropriate animal models which can be used to support pre-clinical efficacy studies.

ZIKV is a mosquito-borne virus belonging to the *Flavivi*rus genus of the *Flaviviridae* family and was first isolated in the Zika forest region of Uganda from the blood of a sentinel rhesus macaque [9]. Since that time, the virus has spread from Africa into Asia with little incidence until 2007 when it was associated with an outbreak characterized by rash, conjunctivitis, and arthralgia on the Island of Yap in the Federal States of Micronesia [10]. This outbreak was followed by an epidemic of ZIKV infection in French Polynesia in 2013 and 2014 that was correlated retrospectively with a 20-fold higher incidence rate of GBS [11,12]. A retrospective case-control study confirmed the association of ZIKV with increased incidence of GBS [13], and a second study identified an increased incidence of congenital cerebral malformations in fetuses and newborns associated with ZIKV infection during this time [14]. Subsequent ZIKV outbreaks were reported from numerous islands in the Pacific prior to its emergence in Brazil in March of 2015 [15,16]. As of March 2017, the World Health Organization Situation Report identified 84 countries, territories and subnational areas with evidence of vector-borne ZIKV transmission; 31 countries or territories who have reported microcephaly and other central nervous system malformations possibly associated with ZIKV infection; and 33 countries or territories who have reported and increased incidence of GBS potentially associated with ZIKV infection [17].

Prior to 2015, animal models of ZIKV infection were limited. However, with the increased magnitude of the recent ZIKV outbreaks and severity of clinical disease in both adults and in the developing fetus, there has been a heightened interest in developing animal models to better understand the pathogenesis of different geographic and temporal ZIKV isolates [18]. Recent research efforts with mice have focused predominantly on the use of immunocompromised strains which lack receptors for type I interferon (IFN α/β), type II interferon (IFN γ) or which lack other components of the innate antiviral response [19,20,21,22,23]. Infection of immunocompromised mice with ZIKV is frequently lethal depending on the specific immune deficiency; however, most of these studies used different ZIKV strains, doses, and routes of administration, making it difficult to draw conclusions. While a lethal challenge model provides a definite endpoint for efficacy studies, a central tenant to animal model development under the animal rule (21 CFR Parts 314.600–314.650 and 21 CFR Parts 601.90–601.95) is that disease progression in the model recapitulates that observed in humans. The immunocompromised nature of these models also limits their utility for vaccine efficacy studies. Although no overt disease is observed in wild-type immunocompetent mice challenged with ZIKV, viral RNA in addition to infectious viruses can be detected in serum and tissues depending on the ZIKV isolate and the route of inoculation, and these models have been used for vaccine efficacy studies [19,23,24,25].

Non-human primates (NHP’s) have also been studied extensively following the emergence of ZIKV in the America’s, and rhesus macaques are an obvious first choice given the fact that the virus was originally isolated from this species in 1947 [9]. Consequently, several different groups have evaluated ZIKV pathogenesis in rhesus macaques and the resultant model has been used for vaccine efficacy studies [26,27,28,29,30,31]. Cynomolgus macaques and pigtail macaques have also been evaluated [28,32,33] and all species have been found to be similarly susceptible and affectively recapitulate the human condition; however, each of these studies utilized different ZIKV isolates of either African or Asian lineages, different routes of inoculation, and different challenge doses, again making it difficult to make comparisons. The objective of this study is to provide a definitive evaluation of ZIKV natural history utilizing highly characterized virus stocks of African and Asian lineages to establish an Indian rhesus macaque (IRM) model for product evaluation under the animal rule. In the process, significant differences in the infectivity of IRMs to ZIKV strains of Asian and African lineages were noted and are contrary to previous reports with other strains of the African lineage [34]. The implications of these differences are discussed below.

## 2. Materials and Methods

### 2.1. Care and Use of Animals

This study was designed to use the fewest number of animals possible, consistent with the objective of the study, the scientific needs, contemporary scientific standards, and in consideration of applicable regulatory requirements. The study design was reviewed by the Institutional Animal Care and Use Committee (IACUC) at Southern Research (ACUP#16-08-037F, approved 13 October 2016). Animals were socially housed during the quarantine and pre-study phases, then single housed following challenge phases of the study. Animals were housed in stainless steel cages that meet requirements as set forth in the Animal Welfare Act (Public Law 99–198) and the *Guide for the Care and Use of Laboratory Animals* (8th Edition, Institute of Animal Resources, Commission on Life Sciences, National Research Council; National Academy Press; Washington, DC, USA; 2011). Animals were housed in an environmentally monitored and ventilated room. Fluorescent lighting provided illumination approximately 12 h per day to simulate the natural diurnal lighting and minimize housing-associated stress.

### 2.2. Viruses, Cell Culture

Vero cells were grown in Dulbecco’s Minimal Essential Medium (DMEM, Lonza, Walkersville, MD, USA), supplemented with 10% Fetal Bovine Serum (FBS), NEAA and L-Glutamine according to standard culture conditions. ZIKV strain PRVABC59 was isolated in 2015 from human serum collected in Puerto Rico and obtained from the Centers for Disease Control and Prevention (Division of Vector-borne Infectious Diseases, CDC, Fort Collins, CO, USA). The PLCal_ZV strain (NR-50234) was isolated from a human who had traveled to Thailand in 2013 and was obtained through the Biodefense and Emerging Infections Research Resources Repository (BEI Resources, Manassas, VA, USA), National Institute of Allergy and Infectious Diseases (NIAD), of the National Institute of Health (NIH). ZIKV, IbH_30656, NR-50066 was obtained through BEI Resources, NIAID, NIH, as part of the WRCEVA program and was isolated from human blood collected in Nigeria in 1968. Master and working stocks of each virus strain were amplified in Vero cells (with minimal passages after receiving them in house from the primary source) and quantified using standard plaque assay on Vero cells to determine the plaque forming unit (PFU) titer. All stocks were determined to be free of mycoplasma using a MycoAlert Mycoplasma Detection Kit (Lonza, LT07-118). Endotoxin levels for stocks were determined by a QCL-1000 kit (Lonza, 50-647U) and found to be <0.1 EU/mL. Sterility was also verified by both blood agar and potato dextrose slant cultures for at least 14 days.

### 2.3. Natural History Study

A total of 36 IRM’s seronegative by ELISA for Dengue, West Nile and ZIKV were subdivided into nine groups, each group containing 2 males and 2 females assigned randomly based on body weight. Animals weighed between 3.0 to 7.5 kg and 2–5 years age at the study start.

**Figure 1:**
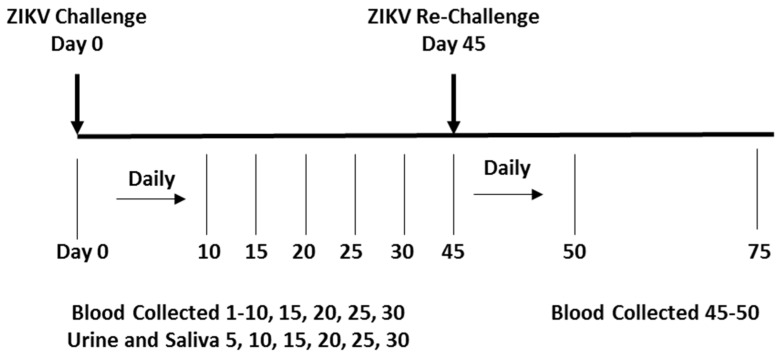
Key Study Challenge and Sample Collection Time Points. Challenge Phase I animals were subdivided into cohorts and inoculated on day 0 with a primary challenge ZIKV isolate. After the primary challenge and a resting period, animals were regrouped and challenged on day 45 with either the same isolate or cross-challenged with an isolate of different geographic origin. Blood, saliva and urine samples were collected as indicated for viral load analysis by qRT-PCR or plaque assay.

For the initial challenge, animals were inoculated subcutaneously (SC) with a ZIKV isolate from either the Asian-lineage (PRVABC59 or PLCal_ZV) or the African-lineage (IbH_30656) at the indicated doses (Table 1). Biological fluid samples (blood, urine, and saliva) were collected between days 0–30 post initial challenge (Figure 1). Following a 2-week resting period, the 12 animals originally challenged with each isolate in Phase 1 were distributed into two groups composed of one male and one female from each low, medium and high dosing group and re-challenged at a dose of 1 × 10^6^ PFU/animal using either the identical strain they were previously exposed to or a different strain as indicated in Table 2. Serum collection during the re-challenge phase paralleled the periodicity of the collection time points of the initial challenge phase, between days 45–75 as per Figure 1.

### 2.4. Primers and Probes

PCR primers and probes were designed to the viral Envelope region of each ZIKV genome, proximal to those described in [35] utilizing modified sequences optimizing primer/probe binding for strains PRVABC59 (Genbank Accession KU501215.1), PLCal_ZV (Genbank Accession KX694532.1) and IbH_30656 (Genbank Accession HQ234500.1). All genomic ZIKV strain analysis used for primer and probe design for PRVABC59 and IbH_30656 were as previously described [36]. For the PLCal_ZV strain, we used the previously described primers Zika Dual-For and Zika Dual-Rev [36] and a sequence optimized probe (5′6FAM-TGC-CCA-ACA/ZEN/CAA-GGC-GAA-GCC-TAC-CT-3′IABkFQ). The probe contains a 5’-6FAM reporter, an internal ZEN quencher and a 3′ IBFQ Iowa Black quencher. All primers and probes were synthesized by Integrated DNA Technologies (IDT, Coralville, IA, USA). Primer and probe combinations were fully characterized for compatible melting temperatures (Tm), self-dimer and hairpin potential as previously described [32]. Primers and protocols used for the generation of the RNA template used for the standard curve for absolute quantitation were as described in [36].

### 2.5. Biological Sample Collection

Biological fluid samples (serum, urine and saliva) were collected from anesthetized IRM at multiple times after the initial challenge on days 0–30 and re-challenge on days 45–79 (Figure 1). Briefly, for the initial challenge (day 0), blood was collected daily (day 0–10) and then on days 15, 20, 25 and 30. After the re-challenge on day 45, blood was collected daily from days 45–55 and thereafter on days 60, 65, 70 and 75. Collected blood samples were immediately processed to serum using serum separator tubes (SST) with brief centrifugation and stored at or below −70 °C. Urine was collected by either cystocentesis (needle) or catheter. Saliva (or drool) was collected directly; if saliva could not be collected, oral cavity swabs were taken and immersed in 1 mL DPBS without Calcium and Magnesium (Cat. # 17-512F, Lonza Walkersville, MD, USA). Both urine and saliva collections occurred on the same schedule, post initial challenge on days 5, 10, 15, 20, 25, and 30 and post re-challenge on days 50, 55, 60, 65, 70 and 75. Upon collection and the preparation of small aliquots, urine and saliva were frozen at or below −70 °C.

### 2.6. Viral Load by qRT-PCR

Viral RNA was extracted from collected biological fluids to quantify viral load. Briefly, using the QIAmp Viral RNA Mini kit, (Qiagen, 52906, Germantown, MD, USA) total RNA was extracted and purified from a biological sample volume of 140 µL (as per the manufacturer) and eluted into 60 µL of nuclease-free water (Ambion, AM9939). Five µL of purified RNA from each test article was used in a 20 µL qRT-PCR reaction consisting of Fast Virus 4× Master Mix (Applied Biosystems, 4444436, Foster City, CA, USA) containing 500 nM forward and reverse primers with a 200 nM probe. Cycling parameters for the QuantStudio Flex 6 instrument include: an initial reverse transcription (RT) step for 5 min at 53 °C, followed by 1 min at 95 °C and 45 cycles of 2-step cycling at 95 °C for 5 s and 60 °C for 50 s. A standard curve for absolute quantitation using a positive control RNA template was established over the dynamic range of 6-logs (1 × 10^6^–1 × 10^1^) with each dilution in triplicate of test samples. As reported in [32] the *C*t values obtained for each of the 6-log dilutions for each strain specific primer/probe combination performed consistently (within 1 *C*t) with each other. NCBI Sequence alignment was used for the development of primers and probes that would work specifically with IbH_30656 and other strains used in this study. Additionally, the lower limit of quantitative detection (LLOQ) is approximately 10 copies/20 µL PCR reaction. Depending on the volume/weight of the extracted test sample and the elution volumes, the resulting dilution factor is typically between 80 and 100 fold, thus the LLOQ for this assay is recorded as 500 copies/mL.

### 2.7. Viral Load by Plaque Assay

The 3 serum samples from each primary and secondary challenge containing the highest viral load as determined by qRT-PCR were further assessed by plaque assay on Vero cells. Briefly, Vero cells were cultured in 6-well plates to approximately 90–100% confluent monolayers and cells were exposed to 200 µL of 4-fold serially diluted samples. Three dilutions of each sample were tested in duplicate. Plates were incubated at 37 °C and 5% CO_2_ for 1 h before the addition of an overlay media consisting of EMEM and 0.5% agarose. Plates were incubated for 3 to 5 days until discernable plaques were formed then fixed and stained with crystal violet.

### 2.8. Indirect ELISA

Purified African lineage ZIKV lysate (The Native Antigen Company, Oxfordshire, UK) was diluted to 0.5 μg/mL in carbonate-bicarbonate buffer (Thermo Fisher Scientific, Waltham, MA, USA). Lysate was generated by culturing virus in Vero cells, clarified, concentrated via sucrose gradient ultracentrifugation, then lysed in Triton X-100, and heat-inactivated. Use of a whole virus lysate provides magnitudes of additional linear epitopes, when compared to use of a single viral protein (Env or NS1 only). This allows for increased epitope presentation, presumably including targets present on both African and Asian lineages.

High binding MAXISORP™ 96-well plates (Thermo Fisher Scientific) were coated with 100 μL of diluted antigen and refrigerated at 2–8 °C overnight. Plates were washed five times with 0.05% Tween 20 in PBS, then blocked for 30 min at 37 °C using 5% dry milk (Quality Biological, Gathersburg, MD, USA). Plates were washed five times then incubated for 1 h at 37 °C with serially diluted sera samples. Signal was detected using goat anti-human HRP conjugated IgG (SeraCare, Milford, MA, USA) diluted 1:2000 in 5% milk. Following washing, 100 μL of ABTS 1-component peroxidase substrate (SeraCare, Milford, MA, USA) was added to each well under low light. Plates were covered and incubated at room temperature for fifteen to twenty minutes, then 100 μL of 1% sodium dodecyl sulfate (Fisher Scientific) was added to stop the reaction. Absorbance optical density (OD) was read at 405 nm on a SpectraMax i3 using Softmax Pro software (Version 6.3 GxP, Sunnyvale, CA, USA). Averaged OD values were plotted against the log of the reciprocal of their dilution (ex. Log_10_100, Log_10_12800). A four-parameter non-linear regression method (GraphPad Prism 5 software, La Jolla, CA, USA) was used for data analysis. Using the formula from curve fitting, data points between each dilution step were extrapolated, expanding the data from eight to one thousand OD values. The log of the reciprocal dilution that corresponded to the extrapolated OD value at a cut-point was identified. The antilog of each identified value at the cut-point was calculated. This value is the reciprocal of the lowest serum dilution capable of producing a positive result for anti-ZIKV IgG antibodies.

An endpoint cut-off was defined as follows: more than twenty IRM serum samples were tested using commercially available kits for ZIKV (XpressBio, Frederick, MD, USA) and Dengue Virus (Calbiotech, El Cajon, CA, USA and Abcam, Cambridge, MA, USA). Sera testing negative for both viruses on all kits were run on the above ELISA, with the exception that samples were diluted 1:100 only. Naive sera averaged an OD value of 0.064. The cut point OD value (0.148) was calculated by adding two times the standard deviation of the OD values to the average OD value.

### 2.9. Focus Reduction Neutralization Test (FRNT)

The FRNT assay for ZIKV was performed on serum samples collected at the time points indicated. Briefly, Vero cells seeded at a concentration of approximately 1 × 10^5^ to 2 × 10^5^ cells/mL in a 96-well plate were incubated for approximately 24 h. On the day of the assay, input virus and serially diluted serum samples were mixed and incubated for 1 h at 37 °C ± 1 °C in the dilution plate. The supernatant was decanted from cell-seeded 96-well plates and then 100 µL of virus/serum mixture was transferred from the dilution plate and added to the cells. After adsorption for 1 h, overlay medium was added to the plate and incubated at 5% CO_2_ overnight. The following day, the plates were stained using broad spectrum pan-flavivirus antibody (MAB10216, Millipore, Burlington, MA, USA) and Goat anti-mouse IgG (H + L) HRP-conjugated secondary antibody (5220-0341, SeraCare Life Sciences, Milford, MA, USA). TrueBlue Peroxidase Substrate (5510-0030, SeraCare Life Sciences, Milford, MA, USA) was added to the plates and spots were analyzed by BioSpot scanner. Each dilution of sera was tested in triplicate. Neutralizing antibody titers were reported as the inverse of the serum dilution estimated to reduce the number of input virus by 50% (FRNT_50_). The percent neutralization at each dilution was calculated by the ratio of average foci counts of the replicates to the average foci of the input virus wells. FRNT_50_ titers were estimated by point-to-point linear regression between the two dilutions that spanned 50% neutralization.

### 2.10. Efficacy and Immunogenicity Testing of Inactivated Vaccine

Formalin Inactivated PRVABC59 Vaccine (lot number 2016) [37], with a concentration of 5 µg/0.5 mL, obtained from Walter Reed Army Institute of Research (WRAIR, Springfield, MD, USA) was used for the study. Prior to Study day 0, four (4) IRMs were randomized into two groups (2 animals/group) according to gender/weight using Provantis Software. On days 0 and 28, all animals were anesthetized and inoculated intramuscularly (IM) with inactivated-PRVABC59 vaccine (Group 1) or SC with Minimal Essential Medium supplemented with 1% FBS as a vehicle control (Group 2). On day 56, all macaques were anesthetized and challenged SC with 0.5 mL of ZIKV strain PRVABC59 with a target challenge dose of 1 × 10^5^ PFU per animal. Blood samples were collected on day 0 (prior to immunization), day 14, day 28 (prior to boost) and day 56 (prior to challenge) to determine anti-ZIKV neutralizing antibody (Nab) titer’s by FRNT. Viral load was assessed by qRT-PCR on serum samples collected daily between days 56 and 66 and again on day 85.

## 3. Results

### 3.1. Clinical Observations

All of the animals were monitored twice daily during the challenge and re-challenge phases. With the exception of some mild erythema, described below, none of the animals showed overt signs of clinical manifestations associated with ZIKV infection throughout the study. On day 1, ZIKV challenge-associated mild erythema at the injection site was observed in 5 animals; however, there was no correlation with challenge dose or strain, and lesions resolved by day 2. Mild erythema at the inguinal region was also observed in one animal challenged with PLCal_ZV at 1 × 10^6^ PFU on day 6 and another challenged with the PRVABC59 strain at 1 × 10^5^ PFU on day 21. Mild erythema was detectable for only one day and lesions were barely perceptible in these monkeys. ZIKV re-challenge-associated mild erythema at the injection site was observed in a total of 13 animals distributed throughout the study groups on day 46 and one animal on day 47, but resolved afterwards without progressing further. Overall, mild erythema detected post primary and secondary challenge was mostly limited to the local injection site and completely resolved without desquamation.

### 3.2. Virus Detection Following Primary Infection

**Figure 2:**
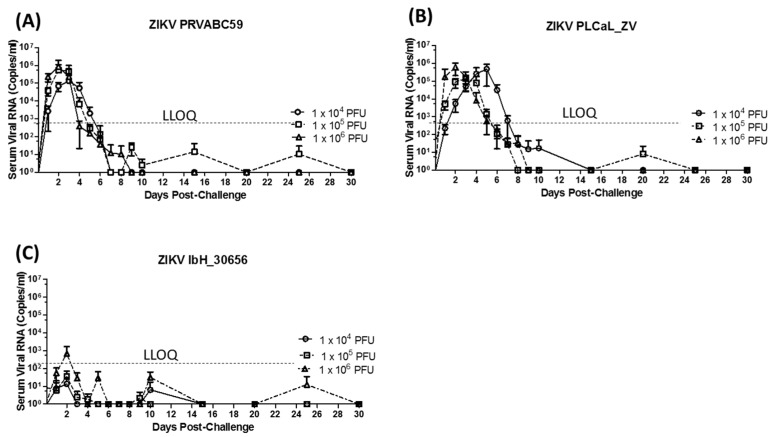
Serum viral RNA copies in IRMs infected with different ZIKV isolates: Serum viral RNA copies in the IRM’s (*N* = 4 per dose group) infected with different doses of ZIKV isolates as indicated in the figures. (**A**) PRVABC59 (**B**) PLCal_ZV (**C**) IbH_30656. Viral RNA is reported as copies/mL from serum purified from blood collected at multiple time points post challenge between days 0–30. Lower Limit of Quantitation (LLOQ) is shown by the dotted line.

Following the challenge, the dose preparations were back titered via plaque assay, and targeted doses were confirmed for all but the high, medium and low dose groups for IbH_30656 and the low dose group for PLCal_ZV, which were slightly more than 0.5 logs below the targeted dose (Table 1). Serum, saliva and urine samples collected at the time points indicated in Figure 1 were tested by qRT-PCR to estimate the relative level of virus replication in each animal. Analysis of the serum viral RNA load (Figure 2) showed that animals challenged with PRVABC59 developed peak concentrations on day 2 in the mid and high dose groups as compared to day 3 in the low dose group (Figure 2A). Regardless of the dose concentration, viral RNA fell below the LLOQ after day 4 in serum from animals challenged with PRVABC59. Animals challenged with PLCal_ZV demonstrated similar serum viral RNA kinetics to PRVABC59 at the mid and high dose levels, but peak RNA concentration was delayed until day 4 and persisted until after day 6 in the low dose group (Figure 2B). Animals challenged with the IbH_30656 (African) strain demonstrated only low levels of viral RNA in serum samples collected throughout the study that were generally at or below the LLOQ (Figure 2C). Only the highest dose group produced an average viral RNA concentration above the LLOQ on day 2 that was transient.

Serum samples with high viral RNA concentrations in the qRT-PCR assay (above the LLOQ) were selected for testing by standard plaque assay to quantify infectious virus particles present in serum, and the comparative results are presented in Tables S1 and S2. Based on these criteria, the viral plaque assay was only performed on samples from IRMs challenged with PRVABC59 and PLCal_ZV. Infectious virus particles were detected in most samples confirming viremia; however, viral PFU titers were generally 2 to 3 logs lower as compared to viral genome levels. In some cases, no plaque titers were detected despite RNA copy numbers as high as 1.12 × 10^5^/mL; thus, there was no consistent correlation between RNA copy number and plaque titer, and qRT-PCR analysis was confirmed to be a more reliable method for the detection of the virus in serum. 

**Figure 3:**
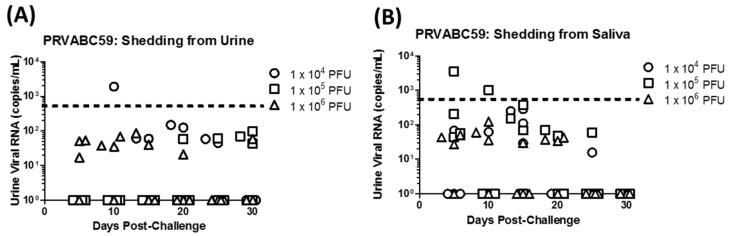
Viral RNA shedding in PRVABC59 infected IRM: Urine and saliva were collected at multiple time points post challenge between days 0–30 from IRM infected with ZIKV PRVABC59 and subjected to qRT-PCR assay. ZIKV RNA concentrations (copies/mL) in the urine (**A**) and saliva (**B**). Circles represent data from single animals in the 1 × 10^4^ dose group, squares represent the 1 × 10^5^ dose group, and triangles represent the 1 × 10^6^ dose group. Urine could not be collected from one IRM on day 5 (dose 1 × 10^4^) and day 25 (dose 1 × 10^5^). Lower Limit of Quantitation (LLOQ) is shown by the dotted line.

Detectable levels of viral RNA were also observed in urine and saliva samples collected throughout the study from animals challenged with the PRVABC59 and PLCal_ZV strains. However, RNA detection from individual animals was sporadic and independent of dose concentration as demonstrated in Figure 3 for animals challenged with PRVABC59. In this case, low levels of viral RNA (5.3 to 69 copies/mL on average) were detected in urine samples up to day 30 (Figure 3A), yet only one animal challenged with the lowest dose had RNA levels above the LLOQ on day 10 post infection. Viral RNA ranging from 3.9 to 970 copies/mL on average was also observed in saliva samples up to day 25, but again only two samples from animals in the medium dose group had detectable RNA above the LLOQ on days 5 and 10 post-infection (Figure 3B). Results were similar for samples collected from animals challenged with PLCal_ZV; however, viral RNA was only detected in 4 urine samples from animals challenged with IbH_30656 collected over the course of this study while none of the saliva samples had detectable RNA levels [38]. The most notable observation from the analysis of urine and saliva samples from animals challenged with Asian lineage viruses is that viral RNA is most reliably detected (though below the LLOQ) in saliva samples collected 5 days post infection as demonstrated for PRVABC59 in Figure 3.

### 3.3. Immune Response Following Primary Infection

**Figure 4:**
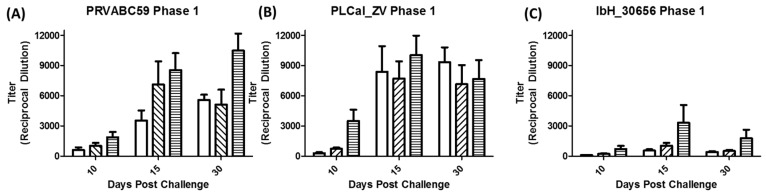
Anti-ZIKV IgG production after initial challenge with ZIKV. Animals were challenged with 1 × 10^4^ PFU, 1 × 10^5^ PFU, or 1 × 10^6^ PFU with either ZIKV (**A**) PRVABC59, (**B**) PLCal_ZV or (**C**) IbH_30656 and anti-ZIKV IgG was detected in serum by ELISA. Bars with no fill 1 × 10^4^ PFU, hatched lines 1 × 10^5^ PFU, or horizontal lines 1 × 10^6^ PFU.

Anti-ZIKV antibodies induced following challenge with varying doses of ZIKV are illustrated in Figure 4 and Table S3. IgG antibodies were measured using an in-house quantitative ZIKV ELISA against a purified African lineage ZIKV lysate and enumerated using four-point non-linear regression analysis. Following the challenge, a dose-dependent anti-ZIKV immune response was detected by day 10 in all groups though higher levels of IgG were observed in IRMs challenged with Asian lineage strains (PRVABC59 and PLCal_ZV) as compared to the African lineage strain (IbH_30656). Antibody titers were weak for IbH_30656-infected animals presumably due to very low infection of the animals. The highest average IgG titers on day 10 were observed in the high dose groups challenged with PLCal_ZV, followed by PRVABC59 and IbH_30656. By day 15, antibody titers in the high dose groups of each isolate increased; however, by day 30, average IgG antibody titers from IRMs challenged with PLCal_ZV and IbH_30656 began to decrease while PRVABC59 continued to increase. IRMs challenged with PLCal_ZV and PRVABC59 at the median dose level had averaged titers of 732 and 1026, respectively, on day 10 as compared to an average titer of 226 in those challenged with IbH_30656. Titers peaked at this dose level in all groups on day 15. By day 30, PLCal_ZV titers in the median dose group were retained, while PRVABC59 and IbH_30656 titers decreased. In the lowest dose groups, only one of four animals challenged with IbH_30656 and three of four animals challenged with PLCal_ZV had an IgG Antibody titer above the LLOQ, whereas all animals challenged with PRVABC59 at this dose on this day had a detectable titer. By day 15, the average IgG titers increased in IRMs challenged with PRVABC59 and PLCal_ZV and titers continued to increase on day 30. IgG antibody titers in IRMs challenged with the low dose of IbH_30656 also increased on day 15 but fell on day 30.

### 3.4. Virus Detection Following Secondary Challenge

**Figure 5:**
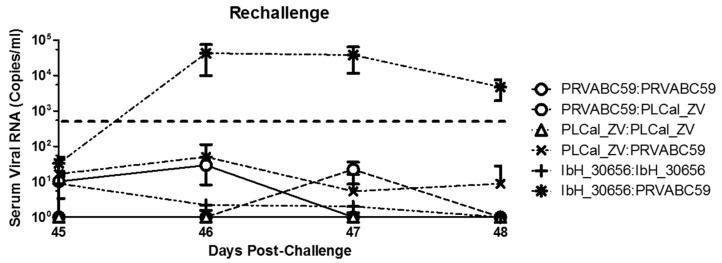
Serum viral RNA copies post rechallenge in IRMs: Serum viral RNA copies were determined by qRT-PCR in IRMs (*N* = 6 per group) previously infected and re-challenged with either PRVABC59, PLCal_ZV, or IbH_30656 at a dose of 1 × 10^6^ PFU/IRM. Viral RNA is reported as copies/mL of serum purified from blood collected at multiple time points post re-challenge between days 45–50. Note, for the IbH_30656:IbH_30656 group day (50) viral RNA copies were determined from *N* = 3. Lower Limit of Quantitation (LLOQ) is shown by the dotted line.

After primary challenge and the conclusion of the natural history study, animals were regrouped as described in the materials and methods section and were challenged on day 45 either with the same isolate they were previously exposed to or cross-challenged with an isolate of different geographic origin (Table 2). The targeted dose of 2 × 10^6^ PFU/mL was confirmed by plaque assay (Table 2), and serum was collected to assess viral load and immunological responses as before (Figure 1). In animals previously challenged with PRVABC59, viral RNA that was below the LLOQ was detected in only 4 samples following re-challenge with PRVABC59 and two samples from animals challenged with PCal_ZV (Figure 5). Following homologous challenge, no viral RNA was detected in serum samples from IRMs previously challenged with PLCal_ZV; however, RNA that was below the LLOQ was detected in at least one animal at various times when challenged with the heterologous PRVABC59. Re-challenging IRMs with IbH_30656 resulted in detectable levels of RNA in multiple samples that were similar to the levels seen following primary challenge but again below the LLOQ. In contrast, challenging IRMs with PRVABC59 following a primary challenge with IbH_30656 resulted in detectable levels of viral RNA that were attenuated by only two logs as compared to naïve animals challenged with PRVABC59 (Figure 5 vs. Figure 2A). In this case, peak concentrations of viral RNA were achieved between days 2 and 3 post challenge and persisted to day 5 before falling below the LLOQ (Figure 4).

### 3.5. Immune Response Following Secondary Challenge

**Figure 6:**
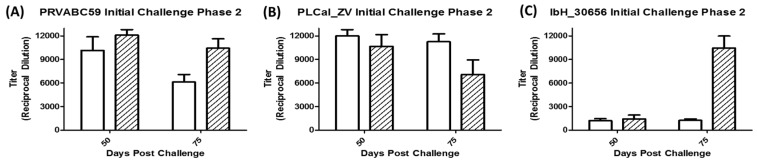
Anti-ZIKV IgG production after secondary challenge with ZIKV. Antibody titers on Study days 50 and 75 during Phase II. Animals from each group in Phase I were reassigned based on previous challenge strain and titer, then re-challenged with 1 × 10^6^ PFU of PRVABC59, PLCal_ZV, or IbH_30656. (**A**) Primary challenge with PRVABC59 followed by secondary challenge with PRVABC59 (No fill) or PLCal_ZV (Hatched lines); (**B**) Primary challenge with PLCal_ZV followed by secondary challenge with PLCal_ZV (No fill) or PRVABC59 (Hatched lines); and (**C**) Primary challenge with IbH_30656 followed by secondary challenge with IbH_30656 (No fill) or PRVABC59 (Hatched lines).

Figure 6 and Table S4 illustrate the anti-ZIKV immune response at 5 and 30 days post-secondary challenge (Study days 50 and 75, respectively) with homologous and heterologous ZIKV challenge. Average IgG antibody titers ranged between 10,141 and 12,099 on day 50 when IRMs received homologous or heterologous secondary challenges with high doses of PRVABC59 or PLCal_ZV. IgG concentrations remained high on day 75 for IRMs that received a secondary challenge with PLCal_ZV (Figure 6A,B), while titers of animals re-challenged with PRVABC59 decreased. Re-challenging IRMs with IbH_30656 did not increase the average IgG immune response on days 50 or 75; however, challenging IRMs with PRVABC59 following an initial exposure to IbH_30656 resulted in increased IgG concentrations on day 75 of 10,456 on average as shown in Figure 6C.

### 3.6. Immunogenicity and Efficacy of Inactivated ZIKV Vaccine

**Figure 7:**
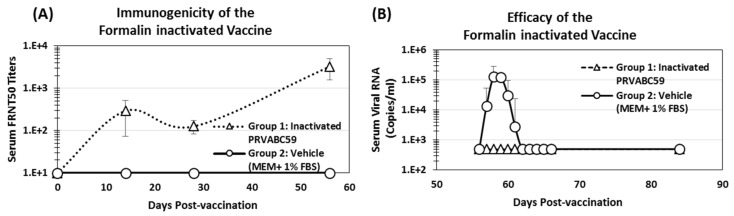
Immunogenicity (**A**) and Efficacy (**B**) of Formalin-inactivated ZIKV vaccine in the IRM model: IRMs were vaccinated with formalin-inactivated ZIKV vaccine or the sham control on day 0, followed by challenge with 1 × 10^5^ PFU of PRVABC59 per IRM on day 54. Serum viral RNA copies after the challenge and FRNT50 titers after vaccination were measured by using qRT-PCR assay and ZIKV neutralization assay as described in the method section. Triangles—Vaccinated animals, Circles—Vehicle control.

To demonstrate the utility of the resultant model, groups of 2 IRMs were immunized twice (day 0 and 28) with inactivated PRVABC59 vaccine (Group 1) or vehicle alone (Group 2) followed by SC challenge with PRVABC59 virus on day 56. Immunogenicity of the inactivated vaccine was evaluated by measuring Nab’s as shown in Figure 7A. FRNTs in sera collected on day 14 demonstrated seroconversion in both animals in the vaccinated group with an average FRNT_50_ titer of 294. By day 28, prior to boost, FRNT_50_ titers had decreased to 127 on average; however, following the boost and prior to challenge on day 56, FRNT_50_ titers averaged 3270 in the two vaccinated animals. In contrast, ZIKV neutralizing antibodies were not observed in the vehicle control animals.

Efficacy of the vaccine was evaluated by assessing serum viral loads in the vaccinated and control animals via qRT-PCR as shown in Figure 7B. Viremia was below the LLOQ in vaccinated animals throughout the study, whereas control animals showed significant viremia starting on day 57 and peaking on day 58. Individual peak viral titers in the unvaccinated animals ranged from 6.2 × 10^4^ to 2.8 × 10^5^ GE/mL and viral titers fell below the LLOQ by day 62, 6 days post challenge, similar to what was observed in preliminary studies.

## 4. Discussion

In 2016, more than 40,000 symptomatic Zika disease cases, not including congenital disease cases, were reported in the United States and its territories alone (https://www.cdc.gov/zika/reporting). In 2017, the number decreased to less than 1000; however, the global incidence remained high with as many as 84 countries, territories or subnational areas reporting evidence of vector-borne ZIKV transmission [17]. In the same report, at least 13 countries have also reported evidence of person-to-person transmission, underscoring the need to better understand the factors contributing to the epidemiology and pathogenesis of ZIKV and to develop new vaccines and therapies to prevent or treat disease in humans. Paramount to addressing these needs are animal models which recapitulate the human condition, and NHPs have arguably proven to be the most appropriate model to fulfill these requirements. The rhesus macaque in particular has been the most extensively utilized to study the pathogenesis of multiple different ZIKV isolates from the Asian-lineage following SC exposure; however, only one isolate of the African-lineage, MR766, has been evaluated via this route to this point [34]. MR766 represents the prototype ZIKV strain originally isolated from rhesus macaques in Uganda in 1947 and was passaged at least 149 times in suckling mouse brains [9]. IRMs challenged with ZIKV strain MR766 via the SC route resulted in plasma viral loads that were similar to those seen in IRMs challenged with a French Polynesian strain (H/FP/2013) isolated in 2013 [34]. Primary infection of IRMs with the MR766 strain also resulted in a robust immune response, which protected the animals from subsequent challenge with H/FP/2013. In a separate study, cynomolgus macaques were demonstrated to be refractory to infection with the IbH_30656 strain of ZIKV which was isolated in Nigeria in 1968 [32]. The IbH_30656 strain falls under the West African subclade of the African-lineage ZIKV isolates as compared to MR766 which falls under the East African subclade [39,40]; thus, it is unclear if the disparity in these results is related to the phylogenetic differences, the extensive passage history of MR766 in mouse brains, or the genetic background of the NHPs. In yet another study, both rhesus macaques and cynomolgus macaques developed detectable viremia following intravaginal (IVAG) and intrarectal (IR) challenge with a ZIKV strain isolated from mosquitoes collected in Senegal in 1984 [29]. This strain (ArD 41525) is also of the West African subclade and closely related to IbH_30656 [39,40], further complicating the interpretation of these results. The IbH_30656 strain was chosen for this comparative study because it is a historical African isolate but unlike MR766 was not passaged in animals with the intent of increasing virulence. The objective of this study was not to provide an exhaustive comparison of Asian and African lineage viruses but to verify previous data with the IbH_30656 strain in cynomolgus macaques and to challenge the dogma from a single study with MR766 that African lineage isolates are equally pathogenic to Asian lineage isolates in rhesus and that an immune response generated to the African-lineage isolates is protective against Asian-lineage viruses. The ultimate goal of these studies was to perform a definitive analysis of ZIKV isolates from Asian and African-lineage using qualified virus stocks and to establish an NHP model of ZIKV suitable for vaccine and antiviral evaluation.

To this end, master and working stocks of ZIKV isolates PRVABC59, PLCal_ZV, and IbH_30656 were prepared in a qualified bank of Vero cells and were confirmed for purity, quantity, mycoplasma contamination, and endotoxin levels. All stocks met the acceptance criteria prior to use in the study. Comparison of the genome copies (as measured by qRT-PCR) and PFU data suggested that generally the genome copies were about 2–3 log higher than the PFU levels. These differences are most likely due to the sensitivities of the assays which are much greater for qRT-PCR that will detect all viral RNA as compared to the plaque assays, which will only detect viable virus able to infect cells in culture. IRMs determined to be naïve to flavivirus exposure were challenged with ZIKV via the SC route and at doses between 1 × 10^4^ and 1 × 10^6^ PFU as has been done previously [27] to most closely mimic the natural route of vector-borne transmission and at theoretical concentrations reported for West Nile Virus in mosquito saliva [41]. More recent studies suggest that the maximum concentration of ZIKV in mosquito saliva is closer to 1 × 10^3^ PFU and that replication kinetics are delayed when ZIKV is delivered via mosquito bite as compared to the SC route at a dose of 1 × 10^4^ PFU [42]. However, the number of mosquitoes feeding on any given animal was not controlled in that study, adding variability to the actual challenge dose, and other aspects of ZIKV infection including tissue distribution were not significantly altered, justifying the dose and route used in these studies. Following the challenge, a transient rash was observed in some IRMs similar to previous reports [34,43]. Previous reports have also reported mild weight loss after infection of IRMs with the French Polynesian strain H/FP/2013 [27] and elevations in body temperatures [26,28]. In contrast, no significant changes in body weight or temperatures were observed in these experiments.

Similar to previously published IRM studies with Asian lineage ZIKV strains and MR766 [27,28,34,44,45], serum RNA levels in PRVABC59 and PLCal_ZV infected NHPs peaked at day 2 to 3 depending on the dose and persisted until day 6). Virus shedding in urine and saliva that persisted to day 30 and day 25, respectively, was also observed similar to previous reports [26,27,43] but at significantly reduced concentrations (1 × 10^2^–1 × 10^3^ genome copies/mL here versus 1 × 10^4^–1 × 10^6^ genome copies/mL in other reports). Differences in virus shedding in urine and saliva might be due to the ZIKV strain used, the method by which the sample was collected, or differences in the sensitivities and detection limits of the qRT-PCR assays, and highlight the need to standardize these methods between laboratories so that accurate comparisons can be made. Regardless, the sporadic nature of virus shedding in urine and saliva reported here and elsewhere limits the utility of these specimens for assessing clinical progression of ZIKV and efficacy of medical interventions as compared to viremia. The immune response to ZIKV PRVABC59 and PLCal_ZV as measured by IgG response was also similar to previous reports [43,44]. All animals challenged with these strains had detectable IgG titers by day 10 that peaked at day 15 or day 30 depending on the challenge dose. Higher IgG titers were generally observed in IRMs challenged with PLCal_ZV as compared with PRVABC59; however, the immune response following exposure to either PLCal_ZV or PRVABC59 was sufficient to protect IRMs from homologous and heterologous re-challenge as demonstrated by serum viral RNA titers that were below the LLOQ.

In contrast to IRMs challenged with PRVABC59 and PLCal_ZV, viremia following primary challenge with ZIKV strain IbH_30656 was below the LLOQ consistent with what was observed previously in cynomolgus macaques [32]. Consistent with the low level infectivity, the immune response to challenge with the IbH_30656 isolate was also low, and although detectable serum IgG responses were observed in all animals by day 15, they were insufficient to protect IRMs from heterologous challenge with the PRVABC59 strain. While these results appear contrary to what was reported previously in IRMs challenged with the MR766 strain, it is most likely that this failure to protect is due to the low level immune response as opposed to the failure of the antibodies to cross protect [34].

These results serve to rule out the genetic background of the NHP model as a factor contributing to the differential infectivity of African-lineage ZIKV strains; however, it is yet to be determined what viral genetic factors contribute to the disparity. Both the IbH_30656 and MR766 isolates have been subject to multiple laboratory passages since their initial isolation more than 50 years ago. Sequence comparisons performed in 2012 demonstrated that MR766 and IbH_30656 differ by 7% at the nucleotide level and 2.2% at the amino acid level, whereas the ArD 41519 strain which was isolated more recently and also productively infected IRMs via the IVAG and IR routes differs from MR766 by 1.7% at the amino acid level despite a 7% nucleotide divergence [40]. These results serve to narrow the amino acid changes to be assessed in future studies aimed at discerning the viral genetic factors that contribute to differential pathologies of ZIKV strains from the African-lineage and should aid in determining the changes that have contributed to recently emerging pathologies associated with Asian-lineage strains.

Until then, the results presented here and elsewhere support the use of IRMs and the ZIKV PRVABC59 strain as a model of Asian-lineage ZIKV infection for the evaluation of new vaccines and therapeutics. The PRVABC59 strain represents a human clinical isolate of low passage which is a prerequisite for animal model development under the animal rule. Additionally, infection of IRMs with as little as 1 × 10^4^ PFU of virus results in detectable viremia that can be assessed by qRT-PCR. Vaccinating IRMs with an inactivated PRVABC59 vaccine resulted in Nab’s of similar titers and kinetics to those reported previously [31], and vaccinated animals were protected from challenge with 1 × 10^5^ PFU of live ZIKV PRVABC59 as determined by the absence of viremia as compared to sham vaccinated animals. This model utilizing qualified master and working virus banks will serve as a valuable resource suitable for submission of preclinical efficacy data on new ZIKV vaccines and therapeutics to the FDA.

## Figures and Tables

**Table 1:** Animal groupings of (36) Indian Rhesus Macaques subdivided into 9 groups for initial challenge with the ZKV isolate at the dose indicated.

Group Number	Animal Number	Isolate	Target Dose (PFU/Animal)	Delivered Dose (PFU/Animal)
1	4 (2M/2F)	PRVABC59	1 × 10^4^	5.5 × 10^3^
2	4 (2M/2F)	PRVABC59	1 × 10^5^	9.1 × 10^4^
3	4 (2M/2F)	PRVABC59	1 × 10^6^	7.6 × 10^5^
4	4 (2M/2F)	PLCal_ZV	1 × 10^4^	3.0 × 10^3^
5	4 (2M/2F)	PLCal_ZV	1 × 10^5^	9.0 × 10^4^
6	4 (2M/2F)	PLCal_ZV	1 × 10^6^	6.5 × 10^5^
7	4 (2M/2F)	IbH_30656	1 × 10^4^	2.8 × 10^3^
8	4 (2M/2F)	IbH_30656	1 × 10^5^	3.4 × 10^4^
9	4 (2M/2F)	IbH_30656	1 × 10^6^	3.9 × 10^5^

**Table 2:** Animal groupings of (36) Indian Rhesus Macaques for the re-challenge phase resulting in some animals being re-challenged with the identical geographical isolate or cross challenged with a ZKV isolate from a different geographical location at the dose indicated.

Group Number	Animal Number	Initial Challenge	Secondary Challenge	Target Dose (PFU/Animal)	Delivered Dose (PFU/Animal)
1	6 (3M/3F)	PRVABC59	PRVABC59	1 × 10^6^	5.9 × 10^5^
2	6 (3M/3F)	PRVABC59	PLCal_ZV	1 × 10^6^	1.4 × 10^6^
3	6 (3M/3F)	PLCal_ZV	PLCal_ZV	1 × 10^6^	1.4 × 10^6^
4	6 (3M/3F)	PLCal_ZV	PRVABC59	1 × 10^6^	5.9 × 10^5^
5	6 (3M/3F)	IbH_30656	IbH_30656	1 × 10^6^	1.0 × 10^6^
6	6 (3M/3F)	IbH_30656	PRVABC59	1 × 10^6^	5.9 × 10^5^

## Supplementary Materials

The following are available online at http://www.mdpi.com/1999-4915/10/5/229/s1.
