# Supplementary material for: Comparative Pathogenesis of Asian and African-Lineage Zika Virus in Indian Rhesus Macaque’s and Development of a Non-Human Primate Model Suitable for the Evaluation of New Drugs and Vaccines
Source: Viruses. 2018 May 1;10(5):229. doi: 10.3390/v10050229 (PMC5977222; doi:10.3390/v10050229)
Supplement: Supplementary file 1 [file viruses-10-00229-s001.pdf]

Supplementary Data:

Table 1. PRVABC59 viral load in serum detected by quantitative RT-PCR (Genome Copies/mL) and Viral Plaque Assay (PFU/mL).

				Day Post Infection							
				1		2		3		4	
Group	Animal	Dose	Sex	RT-PCR	Plaque	RT-PCR	Plaque	RT-PCR	Plaque	RT-PCR	Plaque
1	5366	1.0×10^4	M	-	-	1.63E4	6.25E1	1.80E4	2.50E1	9.70E3	5.75E2
	5362	1.0×10^4	M	6.29E3	2.50E1	8.05E4	3.38E2	6.10E4	2.50E1	-	-
	5388	1.0×10^4	F	-	-	9.60E4	2.50E2	1.12E5	6.25E1	8.82E4	0
	5376	1.0×10^4	F	-	-	8.48E4	3.75E2	3.44E5	5.75E2	1.12E5	0
2	5360	1.0×10^5	M	4.04E4	6.25E1	1.39E6	3.18E3	1.20E6	1.33E3	-	-
	5368	1.0×10^5	M	2.64E4	1.25E2	2.39E5	9.25E2	1.39E5	2.88E2	-	-
	5389	1.0×10^5	F	2.17E4	1.50E2	3.80E5	1.29E3	4.72E5	8.88E2	-	-
	5378	1.0×10^5	F	6.22E4	6.25E1	1.26E5	4.00E2	5.66E4	3.75E1	-	-
3	5374	1.0×10^6	M	2.21E5	1.50E2	6.07E5	1.13E3	-	-	-	-
	5363	1.0×10^6	M	9.02E4	5.00E1	2.34E5	4.38E2	1.19E5	4.00E2	-	-
	5375	1.0×10^6	F	2.41E5	6.25E1	1.47E5	2.13E2	2.54E4	1.25E1	-	-
	5381	1.0×10^6	F	3.63E5	3.75E1	2.43E6	5.75E2	7.80E5	5.38E2	-	-

Table 2. PLCal_ZV viral load in serum detected by quantitative RT-PCR (Genome Copies/mL) and Viral Plaque Assay (PFU/mL).

				Day Post Infection											
				1		2		3		4		5		6	
Group	Animal	Dose	Sex	RT-PCR	Plaque	RT-PCR	Plaque	RT-PCR	Plaque	RT-PCR	Plaque	RT-PCR	Plaque	RT-PCR	Plaque
4	5370	1.0×10^4	M	-	-	-	-	7.53E4	1.00E2	2.00E5	2.50E1	2.83E4	0	-	-
	5359	1.0×10^4	M	-	-	-	-	-	-	2.42E4	0	2.21E5	3.00E2	2.74E4	2.50E1
	5392	1.0×10^4	F	-	-	-	-	3.88E4	0	7.05E5	8.38E2	7.02E5	1.25E2	-	-
	5383	1.0×10^4	F	-	-	-	-	-	-	8.26E4	7.50E1	9.46E5	4.50E2	7.55E4	0
5	5369	1.0×10^5	M	3.11E3	0	5.41E4	6.00E2	7.08E4	2.00E2	-	-	-	-	-	-
	5358	1.0×10^5	M	-	-	5.39E4	1.00E2	2.22E5	2.50E1	2.29E5	2.50E1	-	-	-	-
	5380	1.0×10^5	F	-	-	9.03E4	3.00E2	9.11E4	5.00E1	3.21E4	0	-	-	-	-
	5384	1.0×10^5	F	-	-	1.56E5	1.88E2	1.67E5	6.25E1	5.90E4	0	-	-	-	-
6	5367	1.0×10^6	M	3.86E4	1.75E2	5.90E5	1.55E3	3.57E5	1.00E2	-	-	-	-	-	-
	5372	1.0×10^6	M	4.28E4	5.00E1	2.37E5	7.00E2	1.37E4	1.25E1	-	-	-	-	-	-
	5379	1.0×10^6	F	-	-	4.26E5	1.00E2	9.63E4	1.25E1	2.92E4	0	-	-	-	-
	5377	1.0×10^6	F	6.14E5	1.58E3	1.17E6	1.15E3	2.52E5	2.50E2	-	-	-	-	-	-

Table 3. Zika IgG ELISA data for Phase I of Natural History study

Phase I						Average		
Group	Challenge Dose (PFU/Animal)	Animal ID	Day			Day		
			10	15	30	10	15	30
1	1E4 PRVABC59	**5366**	218.6	1755.4	4126.5	606.4	3558.1	5573.2
		**5362**	1431.5	6357.5	5769.1			
		**5388**	399.1	2866.8	6739.0			
		**5376**	376.5	3252.6	5658.1			
2	1E5 PRVABC59	**5360**	938.2	2866.8	1925.1	1025.9	7121.5	5127.4
		**5368**	1789.8	8888.3	9018.7			
		**5389**	395.3	>12800	5337.8			
		**5378**	980.1	3930.8	4227.9			
3	1E6 PRVABC59	**5374**	2237.8	9421.6	>12800	1890.3	8559.7	10486.3
		**5363**	3174.6	>12800	>12800			
		**5375**	1383.7	5011.2	10741.7			
		**5381**	765.1	7006.0	5603.4			
4	1E4 PLCal_ZV	**5370**	542.0	>12800	>12800	294.9	8379.9	9341.2
		**5359**	<100	4008.0	8104.8			
		**5392**	345.0	>12800	6026.8			
		**5383**	192.6	3911.8	10433.2			
5	1E5 PLCal_ZV	**5369**	1101.3	>12800	>12800	732.3	7714.7	7151.4
		**5358**	633.1	6513.8	5769.1			
		**5380**	714.8	5576.2	5286.2			
		**5384**	480.0	5968.6	4750.5			
6	1E6 PLCal_ZV	**5367**	6837.9	>12800	>12800	3494.1	10039.1	7685.4
		**5372**	2360.6	9987.0	3950.0			
		**5379**	1925.1	4569.5	7572.1			
		**5377**	2852.9	>12800	6419.6			
7	1E4 IBH_30656	**5361**	123.8	732.4	371.1	106.0	562.3	414.2
		**5365**	<100	787.7	623.9			
		**5385**	<100	242.0	444.1			
		**5390**	<100	487.0	217.5			
8	1E5 IBH_30656	**5371**	173.1	1746.9	843.1	226.2	1039.7	520.8
		**5357**	395.3	1019.0	395.3			
		**5387**	227.2	980.1	511.3			
		**5386**	109.1	412.9	333.5			
9	1E6 IBH_30656	**5364**	765.1	3447.8	1664.1	717.2	3330.9	1790.4
		**5373**	1562.3	8263.8	4207.4			
		**5382**	313.1	768.8	885.1			
		**5391**	228.3	843.1	405.0			

Table 4. Zika IgG ELISA data for Phase II of Natural History study

Phase II					Average	
Group	Re-Challenge Dose (PFU/Animal)	Animal ID	Day		Day	
			50	75	50	75
1	1E6 PRVABC59	**5366**	>12800	8104.8	10141.3	6110.5
		**5360**	3009.4	1878.9		
		**5374**	>12800	7833.9		
		**5388**	12731.8	7248.3		
		**5389**	6706.4	4891.0		
		**5375**	>12800	6706.4		
2	1E6 PLCal_ZV	**5362**	>12800	12426.4	12098.5	10423.0
		**5368**	8591.2	7390.4		
		**5363**	>12800	>12800		
		**5376**	>12800	>12800		
		**5378**	>12800	11005.7		
		**5381**	>12800	6115.3		
3	1E6 PLCal_ZV	**5370**	>12800	>12800	11985.1	11247.5
		**5369**	>12800	>12800		
		**5367**	>12800	>12800		
		**5392**	>12800	>12800		
		**5380**	>12800	9106.7		
		**5379**	7910.4	7178.2		
4	1E6 PRVABC59	**5359**	>12800	>12800	10672.3	7093.0
		**5358**	8845.2	4867.3		
		**5372**	3988.5	3009.4		
		**5383**	>12800	3284.3		
		**5384**	>12800	5797.2		
		**5377**	>12800	>12800		
5	1E6 IBH_30656	**5361**	331.9	516.3	1203.5	1245.6
		**5371**	1184.5	1156.1		
		**5364**	2205.5	1860.7		
		**5385**	924.6	1128.4		
		**5387**	1330.9	1219.5		
		**5382**	1243.4	1592.9		
6	1E6 PRVABC59	**5365**	1249.5	>12800	1431.7	10455.7
		**5357**	1184.5	>12800		
		**5373**	3672.5	>12800		
		**5390**	302.6	4459.9		
		**5386**	1370.3	>12800		
		**5391**	811.0	7074.4		

**Initial Challenge**
1e4 PRVABC59
1e5 PRVABC59
1e6 PRVABC59
1e4 PLCal_ZV
1e5 PLCal_ZV
1e6 PLCal_ZV
1e4 IBH_30656
1e5 IBH_30656
1e6 IBH_30656
